# Faculty Educator Perspectives on Curriculum Reform and Student Use of Commercial Learning Resources in U.S. Preclinical Medical Education: A Structured Narrative Review

**DOI:** 10.7759/cureus.107046

**Published:** 2026-04-14

**Authors:** Yasser A Abdul Bagi, Marian Badriyha, Elizabeth Martinez, Sherese Richards

**Affiliations:** 1 Department of Biomedical Education, California Health Sciences University, Clovis, USA; 2 Department of Pathology, California Health Sciences University, Clovis, USA

**Keywords:** curriculum reform, faculty motivation, learning resources, medical education, self-directed learning

## Abstract

Preclinical medical education in the United States is being reshaped by curriculum reform and increasing student reliance on commercial learning resources (CLRs), altering traditional faculty-student dynamics. The purpose of this structured narrative review is to synthesize the existing literature on faculty educator perspectives regarding the intersection of student CLR utilization and institutional curriculum reform in United States preclinical medical education. While prior literature has examined student CLR use and curriculum redesign separately, this structured narrative review synthesizes their interplay through a faculty-centered lens to inform strategies for stronger faculty-student engagement and more effective curricular approaches by institutions. This review synthesizes twenty-one studies examining faculty perspectives on these changes, focusing on teaching adaptation, motivation, and the impact of self-directed learning. The literature suggests that while students turn to external resources for efficiency and board preparation, faculty often experience reduced autonomy and engagement amid standardized curricula and shifting attendance patterns. The findings underscore the importance of involving faculty in curricular decision-making and providing structured support to better align institutional teaching approaches with evolving student learning behaviors.

## Introduction and background

The preclinical medical school learning environment in the United States has been shifting, with one major catalyst driving change within the past decade being the prolific rise in the use of commercial learning resources (CLRs) by medical students [1]. In addition to shifts in student learning behaviors, medical schools and accreditation bodies have introduced ongoing changes towards a more learner-centered preclinical curricula and pedagogical approaches [2]. Although teaching faculty are the frontline mediators of change in curriculum delivery, they face challenges adapting to the student-level and institution-level changes [3]. As such, an important consideration in assessing CLR utilization trends and institution-level paradigm shifts in preclinical medical education is the faculty perspective. The purpose of this structured narrative review is to examine: How do preclinical medical educators perceive and respond to the concurrent rise in student CLR utilization and institution-level curriculum reform in United States medical schools?

Existing literature has described student patterns of CLR use and curriculum redesign in isolation; this structured narrative review contributes to new insight by synthesizing the interplay of these developments via a faculty-centered lens. By foregrounding faculty experiences, the review aims to inform strategies that strengthen faculty-student engagement and support more effective implementation of institutional approaches to pedagogy and curriculum.

Commercial learning resources (CLRs)

CLRs exist predominantly outside an institution's formal curricular resources (FCRs), usually purchased by medical students to satisfy various learning needs [1]. Some students view CLRs as a more efficient and effective learning modality, enabling them to focus on individual priorities such as hobbies or research [4] or deal with the stressors inherent to medical education, such as the Comprehensive Osteopathic Medical Licensing Examination of the United States (COMLEX-USA) and United States Medical Licensing Examination (USMLE) national licensing examinations [5]. Technological advancements have offered medical students more independent access and greater ownership over choosing learning resources, opening the avenue for individual purchase of CLRs [6].

Today, the CLRs that medical students most often utilize can be categorized as review books, lecture videos, question banks, and repetition/retention tools [7]. The most popular review book is "First Aid for the USMLE Step 1," an 800+ page book with "high-yield" facts, mnemonics, and clinical images [8]. Pre-recorded lectures with figures and animations are a mainstay of the CLR industry, with companies like Pathoma, Sketchy, Boards & Beyond, and Osmosis all producing competing lecture products [9-12]. Question banks (i.e., TrueLearn and U-World) provide students with board-relevant multiple-choice practice items to test their understanding [13]. Memory retention tools include Anki spaced-repetition flashcards for retaining information [14]. The variety of CLRs available allows students to mix and match based on learning modality, in-house curricular demands, and/or board studying [1,6].

The recent untethered access to artificial intelligence (AI) and chatbots such as ChatGPT (OpenAI, San Francisco, CA, USA) represents the next iteration of CLRs [15]. These systems have been shown to excel in a wide breadth of content knowledge and assessment tasks in medical education [16,17]. Current adoption of AI tools by medical students includes content review, self-assessment, and enhanced study sessions with quick answers to select points of confusion [18]. The AI learning tools are a new facet in preclinical medical education, and education in general, pushing student learning further away from FCRs. Faculty educators also have access to the same AI tools, and some are already using them to create or reshape their content and delivery of curriculum in medical education [18].

Evolution of medical education at the institutional-level

Changes in curricular structure and delivery have been ongoing for decades in preclinical medical education, with teaching institutions continuously refining their approaches [2,19-21]. Across the board, classroom instructional methods are steadily shifting to collaborative and learner-centered approaches such as team-based learning (TBL) [22] to promote active learning, which is thought to be superior to lecturing alone [23]. Blood et al. also note how “schools have moved from a decentralized, department and discipline-based structure toward more integrated curricula with centralized administrative oversight” [2]. In other words, preclinical medical education continues to trend toward standardized top-down curriculum and assessment delivery, reducing the autonomy of faculty educators [3]. A nationwide survey of medical school deans showed that half of them are open to the idea of implementing a universal preclinical lecture curriculum [24]. The goal here is not to weigh the advantages or disadvantages of these changes, but rather to contextualize this review’s central focus: their impact on preclinical educators.

The impetus for curriculum changes stems from medical schools and accrediting bodies alike. Self-directed learning (SDL), where the learner is encouraged to independently take initiative in parts of their education, has been more formally emphasized in medical education [25]. The Commission on Osteopathic College Accreditation (COCA) and Liaison Committee on Medical Education (LCME), accrediting bodies for US osteopathic and allopathic schools, started explicitly requiring SDL as an accreditation standard in the past decade [26-28]. This comes during the same period where medical students are increasing their utilization of CLRs, further prompting the need for faculty perspectives on the more autonomous student.

Successful implementation of curricular changes logically necessitates buy-in, expertise, and exposure by educators as they facilitate these recommendations and requirements [29]. However, curricular changes are often dictated at the institution or national level with inadequate faculty training or communication, leaving educators scrambling to adapt or simply reverting to what they are comfortable with [30].

Evolution of medical education as mediated by medical students

While fundamental shifts in the preclinical curriculum have been long-planned and implemented formally at the institutional level, changes that have come about in the past decade related to technology and the subsequent rise in CLRs have occurred more spontaneously and informally by students [1]. Particularly in institutions with a pass-fail curriculum, there is a subset of students who navigate the preclinical years by mainly relying on CLRs over FCRs, including skipping lectures and foregoing faculty-curated content, essentially creating a self-curated parallel curriculum adjacent to an institution’s formal curriculum [1,31].

As students shift their reliance away from FCRs towards CLRs, that change is not always clearly communicated to educators. In a survey of medical students and faculty at one US medical school where the use of lecture supplements was assessed, 78.0% (n=81) of faculty believed students used textbooks, in contrast to only 34.0% (n=155) of students reporting such usage [32]. Similar student textbook utilization rates are echoed in Halperin et al., 41.0% (n=560) [7]. Faculty also overestimated the perceived effectiveness of their curated FCRs by students and underestimated students' use of CLRs [32].

Another key factor that can be partly attributed to CLR proliferation is the decline in lecture attendance in medical school [33]. According to a 2024 Association of American Medical Colleges (AAMC) survey (n =11,010), 43.0% of students reported low attendance at in-person lectures [34], with attendance decreasing as board exams approach [35,36]. Students’ calculus to attend class is multifactorial but is influenced by their perception of instructors' pedagogical methods, quality, and organization, as well as the availability of alternatives such as CLRs [37]. Students and teaching faculty also have differences in their outlook on attendance, where students want to be more laissez-faire with attendance requirements, viewing attendance more as a tool for content learning [38]. Whereas faculty have traditionally viewed lecture attendance as important for professional socialization and exposure to informal social curriculum gained only by interacting with peers and faculty on campus [38]. Some learning differences may be explained by generational divides between students and faculty, whose educational experiences often differ as many years separate educators from learners due to the length of training required in medical education [39].

With these disconnects in mind, medical school faculty are voicing how a more self-directed student body, coupled with unfamiliar student-centered classroom environments and new curriculum structures, are elaborating challenges and frustrations [3].

## Review

Materials and methods 

This study was conducted as a structured narrative review to interpret and synthesize diverse literature describing faculty educators' perspectives within the evolving landscape of preclinical medical education in the United States. The search process began on August 10, 2025, and concluded on September 28, 2025. The authors (YA, MB, SR) collaboratively developed search terms by identifying key concepts from the research question and expanding them using synonyms and related terms. The search terms utilized are captured in Table 1.

**Table 1:** Search terms used in the deconstruction technique

Search terms
Third-party resources medical students
Medical school faculty/educator student connection/support
Medical student self-directed learning
Medical student learning methods
Medical school curricula and third-party resources
Medical education and third-party resources
Change in medical education and third-party resources
Medical school faculty change in teaching resources
Pre-clinical/pre-clerkship medical education and outside resources
Medical student online resource
Pre-clinical medical student extracurricular resources
Faculty perspective of third-party resources

All searches were conducted using free-text terms with Boolean operators AND/OR to combine and expand key concepts; MeSH terms were not applied, which represents an additional methodological limitation. Searches were filtered to January 2000-August 2025 to capture the modern preclinical education era, during which significant curricular and technological shifts occurred. Targeted literature searches were conducted in PubMed and Google Scholar; searches were designed to identify relevant and influential literature rather than to achieve exhaustive coverage. Reference lists of key articles were reviewed to identify additional sources not captured by database searches. The authors, YA and MB, conducted the initial literature search and screened all records against the predefined inclusion and exclusion criteria summarized in Table 2. YA and MB independently reviewed titles and abstracts using these criteria, removed clearly ineligible articles, and then assessed the full texts of the remaining studies. Disagreements between YA and MB were infrequent and resolved through discussion, with SR serving as the adjudicating reviewer when needed. Formal inter-rater reliability statistics (e.g., Cohen's kappa) were not calculated. There were no human subjects or ethical concerns; therefore, this study was exempt from IRB approval.

**Table 2:** inclusion and exclusion search criteria for literature review DO: Doctor of Osteopathic Medicine; MD: Medical Doctor

Inclusion criteria	Exclusion criteria
Studies published within the last 20 years	Studies not written in English
Studies involving medical schools in North America	Studies without available full text
Studies assessing non-curricular learning resources or platforms in preclinical medical education	Studies focusing on non-North American medical education (DO or MD)
Studies examining pre-clinical medical education	Studies referring to undergraduate (pre-medical) education
	Studies focused exclusively on clinical clerkship education
	Studies with a small sample size <5
	Studies deemed to have inadequate methodological quality based on reviewer consensus during full-text screening

**Figure 1:**
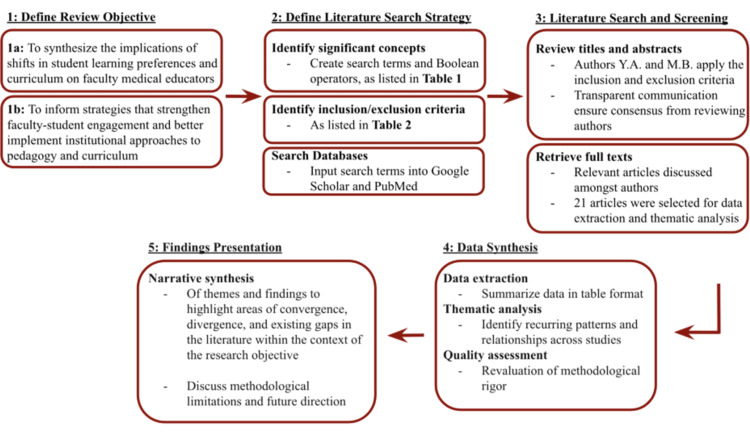
Selection of articles for the literature review

As a structured narrative review, the methodology does not meet the full criteria of a systematic or scoping review, and findings should be interpreted accordingly. Data extraction and synthesis were qualitative and relied on published information, potentially introducing selection and interpretation bias. No formal methodological quality appraisal was conducted, consistent with the interpretive aims of a structured narrative review, potentially introducing subjectivity into the exclusion process. While commentaries and reflective essays represent lower levels of evidence, they were intentionally included where they offered relevant faculty or institutional perspectives not captured by empirical studies alone, given the limited body of primary research directly addressing faculty experiences in this evolving educational landscape. Given the heterogeneity of the study designs included in this review, a standardized risk-of-bias tool was not applied, which represents a limitation of the review's methodology. Data extraction and synthesis were qualitative and relied on published information, potentially introducing selection and interpretation bias. The search strategy was limited to PubMed, Google Scholar, and English-language publications, potentially excluding relevant literature in other databases, gray literature, or non-indexed studies. Reference list searching was performed to partially offset this limitation; however, future reviews on this topic should consider a broader database search strategy. The search strategy and review process flowchart are illustrated in Figure 1.

This search process identified 21 articles for the literature review, examining various facets of faculty and student perceptions, motivations, and outcomes related to teaching, learning resources, and curriculum reform, with methodological limitations summarized in Table 3.

**Table 3:** Studies included in the literature review AAMC: Association of American Medical Colleges, USMLE: United States Medical Licensing Examination, COMLEX: Comprehensive Osteopathic Medical Licensing Examination, MCAT: Medical College Admission Test, DO: Doctor of Osteopathic Medicine, MD: Doctor of Medicine, PharmD: Doctor of Pharmacy, SDL: self‑directed learning, CLR: commercial learning resources

Source (year)	Purpose	Methods	Key findings	Methodological limitations
AAMC, 2024 [34]	To assess preclinical US medical students' perceptions of their preclinical years regarding learning climate, adjustment to medical school, and future plans.	Survey (Likert scale); N=11,122; median response rate 48.9%	43.9% (n=11,010) of pre-clerkship students reported attending in-person lectures 'often' or 'most of the time.' 57.7% (n=10,991) reported virtually attending lectures 'often' or 'most of the time.'	Variations in response rates across medical schools could impact generalizability.
Burk-Rafel et al., 2017 [40]	To examine medical students' study behaviors when preparing for USMLE Step 1 and their association with Step 1 scores.	Survey; N=274; response rate 82.5%; Surveyed population - all medical students at one institution post-USMLE Step 1	Initiating study prior to the designated study period, increased review book usage, and more practice questions were all associated with higher Step 1 scores, even controlling for MCAT scores and preclinical exam performance.	Single-institution study limits generalizability. Cross-sectional design limits causal conclusions. Self-reported retrospective data raises possibility of recall bias.
Campbell et al., 2019 [41]	To characterize faculty perceptions and preferences about student attendance, faculty advising practices, and the impact of low attendance on faculty job satisfaction and teaching.	Survey (Likert and open-ended); N=26; response rate 77%; Surveyed population -medical school faculty including MD, DO, PhD, and PharmD	73% of faculty agreed they felt more job satisfaction with higher student attendance. 58% agreed they would prefer active learning when attendance was mandatory. 46% believed lecture capture was an effective alternative to attending class. 58% disagreed that attendance indicated professionalism.	Self-reported survey data subject to recall bias. Single-institution limits generalizability.
Chang et al., 2022 [42]	To assess pre-clerkship medical students' perceptions of remote learning, exam performance, and CLR utilization during COVID-19.	Survey; N=74; response rate <50%; Surveyed population – preclinical medical students at one institution	Students in remote learning were more likely to feel less connected to peers, report inadequate social interaction, and report weaker peer support systems compared to in-person learning.	Single institution and single cohort limits generalizability. Self-reported data implies recall bias. Response rate <50%.
Hanson et al., 2022 [3]	To assess internal and external motivations of preclinical faculty to continue teaching in the era of curriculum reform.	Survey; N=107; response rate 43%; Surveyed population - faculty teaching pre-clinical curriculum at one institution	Faculty motivation was primarily intrinsic and self-fulfilling. However, external frustrations within and outside the educational realm can interfere with intrinsic motivations and decrease motivation to continue teaching.	Single-institution limits generalizability. Restricting to faculty who taught in the past 5 years may have excluded valuable perspectives on motivation.
Hirumi et al., 2022 [43]	To inform medical educators on students' use of CLRs outside formal curricula, integration of CLRs into curricula, and potential effects on national licensing exam scores.	Narrative literature review	Consistent positive correlations found between question bank use and licensing exam performance. Results provide evidence for integrating CLRs into medical school curricula and highlight the need for further research.	Narrative review is not as methodologically rigorous as a systematic review.
Ikonne et al., 2022 [44]	To assess the frequency of student resource use and academic performance in pre-clerkship education.	Survey; N=88; response rate 54.3%; Surveyed population - preclinical students at one institution	First-year students were more likely to use instructor-produced and self-generated study resources. Second-year students were more likely to use board review resources.	Self-reported survey data subject to recall bias. Single-institution limits generalizability.
Khalil and Kibble, 2014 [30]	To share insights gained during a 4-year journey building a new integrated preclinical medical curriculum.	Reflective essay; single institution	Time spent developing curriculum maps and objectives was less impactful than directing major effort and resources toward faculty development to build integrated sessions with highly engaging teaching methods.	Reflective essay from a single institution; not scientifically rigorous but provides useful institutional insight.
Kortz et al., 2022 [45]	To identify academic and non-academic factors predictive of COMLEX Level 1 scores.	Survey; N=72; response rate ~18%; Surveyed population – third/fourth year medical students who completed COMLEX Level 1 at one institution	After controlling for GPA, three factors explained significant variance in COMLEX Level 1 scores: number of questions completed, number of weeks studied, and money spent on board preparation.	Single institution and single cohort limits generalizability. Self-reported data implies recall bias. ~18% response rate.
Lawrence et al., 2023 [1]	To explore why and how medical students use third-party learning resources.	Qualitative study using grounded theory; virtual focus groups; N=58; response rate 100%; Surveyed population – preclinical medical students from 7 US allopathic medical schools	Students described a cyclical process of hearing about, selecting, and using third-party resources. Reasons included dissatisfaction with institutional curricula, desire for efficiency, and USMLE alignment. Students recommended schools integrate and endorse CLRs.	Qualitative design limits generalizability. Focus on US allopathic schools may not represent all contexts. Self-reported data and participant bias could influence results.
Lau et al., 2025 [46]	To assess the impact and outcomes of a longitudinal faculty development program for physician educators in health systems improvement.	Mixed-methods using Kirkpatrick Model; N=119; Surveyed population - faculty at one US allopathic medical school	A longitudinal, multi-component faculty development program successfully equipped 119 physician educators without prior training to effectively engage medical student-led teams in health systems improvement, with demonstrable faculty satisfaction and student outcomes.	Single institution and single cohort limits generalizability.
Lim et al., 2021 [47]	To assess whether faculty tasked with implementing self-directed learning feel supported by their institutions.	Survey; N=359; response rate 29%; Surveyed population - national sample of U.S. medical educators	94.4% of educators recognized the need for faculty development to enhance SDL, but only 37.5% had received adequate training. Both faculty and students were eager to teach and learn through SDL, but faculty did not always feel sufficiently resourced or supported.	Low response rate; self-reported data.
Makus et al., 2023 [31]	To explore the use of non-traditional resources in undergraduate medical education.	Survey; N=57; response rate 17%; Surveyed population – preclinical medical students at one institution	98.2% of students reported using non-traditional resources. Most common were upper year notes, student-developed Anki decks, and student-developed question banks.	17% response rate may introduce response bias. Self-reported data. Difficulty aligning resources with local exams cited as a limitation. Cost and determining required detail level noted as drawbacks.
Murad et al., 2010 [48]	To determine the effectiveness of self-directed learning in improving learning outcomes in health professionals.	Systematic review; 59 studies; N=8,011 learners; 25 studies (42%) randomized	SDL in health professions education has been shown to be more effective than didactic-based teaching in certain learning domains.	Overall methodological quality of included studies was rated as moderate by the authors.
Parry et al., 2019 [49]	To examine the relationship between CLR use and USMLE Step 1 performance.	Survey; N=170; response rate 70%; Surveyed population – preclinical medical students at one institution	USMLE Step 1 performance was associated with academic performance. A positive association was found between UWorld Qbank use and exam performance. Use of other commercially available products and number of practice tests were not correlated with performance.	Single institution and single cohort limits generalizability. Self-reported data implies recall bias.
Robinson and Persky, 2020 [50]	To discuss the meaning of SDL, challenges with implementation, and strategies to overcome obstacles in educational settings.	Commentary	Care must be taken to appropriately scaffold and structure learning for students new to SDL to develop necessary soft skills. Challenges are faced by both learners and educators when implementing SDL in a classroom setting.	Commentary is not a scientifically rigorous methodology but provides useful contextual insight.
Ruiz et al., 2006 [51]	To introduce e-learning and its role in medical education, including evidence for effectiveness, faculty development needs, and evaluation strategies.	Literature review	Students do not view e-learning as replacing traditional instructor-led training but as a complement forming part of a blended-learning strategy. A developing infrastructure for e-learning includes digital repositories, technical standardization, and peer review methods.	Narrative review is not as methodologically rigorous as a systematic review.
Schick and McWhorter, 2022 [37]	To identify instructor methods and curriculum issues influencing preclinical students' use of scheduled lectures and faculty perceptions of attendance.	Survey; Preclinical students N=144; response rate 47%; Preclinical faculty N=35; response rate 60% at one institution	Students rated ability to explain complex concepts as 'Very Important' for attending lectures. Availability of recorded lectures, proximity to exams, and unscheduled gaps between lectures were key curricular factors. Faculty agreed lectures should continue as a major instructional mode; most spent over 9 hours preparing new lectures.	Self-reported survey data subject to recall bias. Single-institution limits generalizability.
Searle et al., 2011 [52]	To develop recommendations for training faculty who prepare physicians.	Commentary	To change medicine, those who teach medicine must change. This requires specific educational strategies for faculty development and necessitates fundamental shifts in institutional support, including funding, communication, and creation of faculty development networks.	Commentary is not a scientifically rigorous methodology but provides useful contextual insight.
Wong et al., 2021 [4]	To examine medical student learning preferences regarding traditional and third-party resources.	Survey; N=358 (N=224 students, N=133 residents/fellows); Surveyed population – US MD/DO and North American medical schools	Students preferred shorter lectures in the morning, online lectures with animations, and classroom PowerPoint lectures. Students rated third-party resource characteristics higher than traditional curricula.	Unable to synthesize response rate based on distribution method. Data primarily from one institution. Varying education levels of respondents.
Zheng, 2022 [6]	To examine what technologies students adopt for self-directed learning and what factors contribute to self-initiated technology use.	Individual semi-structured interviews; N=26; Surveyed population – preclinical medical students at one institution	Students used four types of technologies: video resources, self-assessment tools, management tools, and social media. Three key determinants of self-directed technology use were identified: perceived usefulness, subjective norms, and educational compatibility.	Small sample from one US medical school limits generalizability. Faculty input was not drawn. Possibility of selection bias in recruitment.

Results

Thematic Analysis of Review Findings 

The data were then analyzed using thematic analysis to identify recurring patterns and relationships across studies. The researchers familiarized themselves with the data, generated initial codes, and identified themes by grouping similar codes. Themes and findings were synthesized narratively to highlight areas of convergence, divergence, and existing gaps in the literature within the context of the research purpose.

Thematic analysis of the 21 included studies identified three recurring themes describing faculty educators' experiences with student use of commercial learning resources and curriculum reform. (1) Medical Student CLR Adoption and Opportunities for Faculty Engagement, supported by studies on medical students' reliance on various CLRs and their perceived effectiveness in enhancing learning. This includes studies assessing the outcomes of utilizing these resources and those seeking to understand their contribution to success on in-house and standardized exams (COMLEX and USMLE). Additionally, the types and categories of CLRs that students rely on and how they select the most beneficial resources, such as content, question banks, flashcards, etc., for different learning needs. (2) Faculty motivation and adaptation in teaching, supported by studies on the internal and external factors that influence faculty motivation and teaching strategies, and the connection to faculty development and faculty satisfaction with increased CLR usage, especially post-COVID. (3) Self-directed learning and curriculum impact, supported by studies looking at the incorporation of self-directed learning into the curriculum and its effect on student attendance, access to materials, and academic performance in internal assessments. This is also supported by studies examining the need for alignment between the institutional curriculum and faculty's preferred resources, and the impact on curriculum design and pedagogical strategies in classrooms. Furthermore, this theme includes institutional support for faculty to implement innovative teaching methods, including the integration of self-directed learning resources.

Discussion

Theme 1: Medical Student CLR Adoption and Opportunities for Faculty Engagement

The increasing reliance on CLRs among preclinical medical students reflects a broader shift in how students engage with learning resources, with meaningful implications for faculty roles. As preclinical medical students progress, particularly as they near board examinations, reliance on FCRs wanes and CLRs gain prominence [40,44]. This trend is propelled by strong correlations between CLR utilization, such as question banks, and improved performance on COMLEX Level 1 and USMLE Step 1 exams [43,45,49]. When students choose not to engage with FCRs or attend class, they easily justify their actions by leaning on CLRs to get them through the content and even perceive that they are making the optimal decision to increase efficiency and customize their learning routine [1,31]. When students prioritize efficiency and self-customized learning routines over FCRs, the reach of faculty-curated content narrows, raising questions about how educators can adapt their role in this evolving environment.

Students have expressed a desire for greater recognition of CLRs by their institutions, advocating for a more integrated approach that combines the strengths of both CLRs and FCRs [1]. This presents an opportunity for teaching institutions and faculty educators to explore and enhance the unique aspects of FCR offerings that are often absent in CLRs. For example, students have indicated a preference for small-group learning, which medical schools provide but CLRs do not [4]. This sentiment was echoed during the COVID-19 remote-learning period, when students felt less connected to and supported by peers [42]. In addition, Zinski et al. found that faculty can help mitigate some of the drop-off in FCR utilization during the second preclinical year by leaning more on clinically oriented teaching methods, such as patient presentations [53]. Across the reviewed studies, findings consistently point to student preference for CLR integration rather than replacing FCRs, suggesting a converging consensus on the complementary roles of these resources. These insights underscore the evolving interplay between students' learning preferences and faculty's teaching practices, as faculty are increasingly challenged to adapt accordingly.

Theme 2: Faculty Motivation and Adaptation in Teaching

To better understand how teaching faculty can accommodate self-directed students in today’s preclinical medical education environment, faculty perceptions should be assessed. Although there is clear educator support for a more self-directed student body, as elaborated by Lim et al. [47], the shift has raised concerns among faculty about their autonomy and the erosion of academic freedom [3]. Faculty members have reported that student attendance and engagement significantly impact their job satisfaction [41], intrinsic motivation to teach [3], and creativity in teaching; factors that are increasingly challenged by current student engagement rates [34]. Additionally, as institutions increasingly dictate changes in curriculum delivery methods (e.g., TBL), faculty often find themselves transitioning between educator and facilitator roles, which requires adjustments in their teaching preparation and approach [3]. These studies suggest that faculty are caught in the middle, balancing evolving demands from institutional curriculum modifications with a more self-directed student body, raising questions about faculty development.

Lim et al. found that while 94.4% (n = 339) of educators recognized the need for faculty development to enhance SDL, only 37.5% had received adequate training [47]. This supports the ongoing need for faculty development, as the existing preparedness of faculty to facilitate SDL is mixed, and educators recognized the need for faculty development yet lacked institutional action [47]. Additionally, educators have decreased intrinsic motivations to teach when gaps in preparation are present [3]. Lau et al. demonstrated the effectiveness of properly implemented faculty development, successfully equipping 119 physician educators without prior training to lead early medical students in health systems improvement projects, with demonstrable faculty satisfaction and student outcomes [46]. While studies converge on the need for faculty development, findings diverge on the extent to which institutional support currently meets that need, reflecting variability across institutional contexts.

In addition to faculty development, the successful implementation of the curriculum developed by planners requires the integration of teaching faculty into the planning process. When a group of curriculum planners reflected on how they built an integrated curriculum for a new medical school, they concluded that their time was better spent “directing a major effort and adequate resources at faculty development” versus developing the curriculum itself [30]. In the context of curriculum development, faculty educators should be placed front and center in the planning process [54]. It would be reasonable to infer that expanding faculty development and exploring the faculty perspective related to the challenges of CLRs and curriculum reform will be essential to fully realizing their potential in preclinical medical education.

Theme 3: Self-Directed Learning and Curriculum Impact

The growing emphasis on SDL in preclinical medical education has complex implications for how students engage with faculty and learning resources. While SDL in health professions education has been shown to be more effective in certain learning domains than didactic-based teaching [48], novice learners, such as first-year preclinical medical students, may have difficulty navigating the various FCRs without proper faculty guidance [50]. Zheng B. found that in an SDL learning environment where the institution implemented a flipped classroom approach, students used more CLRs to facilitate their learning [6]. This example demonstrates an unintended effect: the push for SDL exacerbated the encroachment of CLRs into the formal curriculum.

For SDL to be effective, it requires an institutional environment that nurtures both student autonomy [54] and faculty training to foster confidence in student-centered pedagogy [47]. This necessitates fundamental shifts in institutional support, including funding, communication, and the creation of faculty development networks [52,54]. The introduction of CLRs into medical education contrasts with the earlier integration of e-learning (e.g., personal computers, online modules), which was cautioned to require careful planning and faculty involvement [51]. However, the rapid and decentralized adoption of CLRs today lacks the same level of structured implementation, potentially leading to inconsistencies in educational quality. These findings suggest that the relationship between SDL, CLR adoption, and faculty engagement is not linear, and that institutional efforts to promote student autonomy may paradoxically widen the gap between students and faculty-curated resources. Regardless, there is clear agreement across the reviewed studies that the goal is to create a preclinical learning environment where faculty can help cultivate lifelong learners who can critically assess and integrate diverse learning resources, preparing students for the challenges of medical practice.

Limitations and Future Directions

Common limitations across the studies included reliance on self-reported data, which introduces potential recall bias. Some studies were limited to a single institution or to commentary from a single institution, limiting the broader applicability of the findings. Some studies reported low response rates, which could skew the results and reduce their representativeness. Furthermore, the strength of evidence across the existing studies remains largely limited to single-institution surveys and qualitative designs, underscoring the need for more robust, multi-institutional research. These limitations highlight the need for cautious interpretation and suggest the importance of conducting further research with more diverse and rigorous methodologies. Despite these limitations, the review provides an overview of the current literature on faculty educators' perspectives in the evolving preclinical education environment. As we highlight the use of CLRs by preclinical medical students and explore their broader impacts in the evolving educational landscape, a significant gap in the literature exists regarding faculty and institutional perspectives on CLR use. Future research should examine faculty adaptation to students' use of CLRs to provide a more comprehensive understanding of the implications of CLRs in medical education.

## Conclusions

This structured narrative review identified three principal findings. First, faculty consistently underestimate student CLR use and overestimate student engagement with FCRs, creating a perception gap that affects curriculum design and delivery. Second, faculty motivation and satisfaction decline as student engagement patterns shift, yet institutional investment in faculty development remains insufficient despite widespread recognition of its importance. Third, the push for self-directed learning, while supported by accreditation standards and evidence of effectiveness in certain domains, can unintentionally accelerate CLR adoption and further distance students from faculty-curated content when implemented without adequate faculty preparation.

Across the reviewed literature, a consistent pattern emerges: faculty are navigating simultaneous pressures from top-down curricular standardization and bottom-up shifts in student learning behavior, often without adequate institutional support to do so effectively. The convergence of these pressures has meaningful implications for faculty motivation, teaching autonomy, and faculty-student engagement. Addressing these challenges requires institutions to prioritize faculty inclusion in curricular decision-making and to invest in faculty development programs responsive to the realities of a more self-directed student body. Institutions should aim to offer structured support for both students and faculty to ensure effective integration of CLRs while maintaining educational quality. Future research should continue to explore faculty perspectives on student CLR usage to better inform these institutional efforts.
